# Effects of Attentional Control on Gait and Inter-Joint Coordination During Dual-Task Walking

**DOI:** 10.3389/fpsyg.2021.665175

**Published:** 2021-07-21

**Authors:** Cenyi Wang, Guodong Wang, Aming Lu, Ying Zhao

**Affiliations:** School of Physical Education and Sports Science, Soochow University, Suzhou, China

**Keywords:** attentional control, gait, inter-joint coordination, dual-task walking, performance

## Abstract

In the process of walking, attentional resources are flexibly allocated to deal with varying environmental constraints correlated with attentional control (AC). A dual-task paradigm was used to investigate the effects of AC on gait and inter-joint coordination. Fifty students volunteered to participate in this study. Based on the reaction time (RT) in the Stroop task, the top 15 participants were assigned to the High Attentional Control (HAC) group, while the last 15 participants were assigned to the Low Attentional Control (LAC) group. The participants in the two groups were randomly asked to perform three tasks: (i) single 2-back working memory task (ST 2-back); (ii) single walking task (ST walking); and (iii) dual task (DT). Cognitive outcomes and gait spatiotemporal parameters were measured. Continuous relative phase (CRP), derived from phase angles of two adjacent joints, was used to assess inter-joint coordination. The LAC group exhibited significant task effects regarding RT, correct rate (CR), step width, gait cycle, step time, forefoot contact times, heel-forefoot times, hip-knee mean absolute relative phase (MARP), and deviation phase (DP) in the stance and swing phases (*p* < 0.05). In the HAC group, significant task effects were only detected in RT and foot progression angle of the left foot (*p* < 0.05). Under the three task conditions, the LAC group exhibited a higher CR in ST, longer heel contact times, and longer heel-forefoot times when compared with the LAC group (*p* < 0.05). Compared with the LAC group, the HAC group exhibited significantly smaller (closer to zero) MARP and weaker hip-knee DP values in the swing phase across all gait conditions (*p* < 0.05). In the stance phase, the HAC group had smaller MARP (closer to zero) values when compared with the LAC group (*p* < 0.05). In conclusion, the ability to maintain gait control and modulate inter-joint coordination patterns in young adults is affected by the level of attentional control in accommodating gait disturbances. AC is correlated with the performance of motor control, which theoretically supports the competitive selection of athletes and fall prevention strategies for a specific population.

## Introduction

In human daily life, many activities involve multitasking, which challenges both motor and cognitive functions. Attention plays an essential role in controlling human position and movement; for example, it regulates walking and balance (Woollacott and Shumwaycook, 2002; Möhring et al., 2018). It has been reported that safe walking is a highly attention-demanding task, which requires a high level of mobility skills and cognitive flexibility to attend to a range of environmental demands in order to control movement direction, identify and track visual targets, and be able to read or talk (Lajoie et al., 1993; Buchman et al., 2011). We define attention as the information processing capacity of an individual, which is regarded as limited cognitive resources, for performing tasks affecting the central nervous system (CNS). Therefore, the number of activities that people can perform simultaneously is limited.

Most laboratory studies use the dual-task paradigm to reproduce such daily situations, which is the primary approach to studying the interactions between cognitive processing and motor behavior (Kerr et al., 1985; Abbud et al., 2009; Nordin et al., 2010; Hallal et al., 2013; Patel et al., 2014; Leone et al., 2017). In these studies, walking was performed in tandem with another attention-demanding task, and performance of one or both tasks may be deteriorated (Neumann, 1984; Wickens, 1989; Mcsp, 1996; Shumway-cook and Woollacott, 2000; Leone et al., 2017), which is believed to result from competition for attentional resources (Bynickersonr, 1980) or competition for information processing neural pathways (Pashler, 1994). Gait is a complex process that requires integrating various sensory inputs from visual-vestibular and proprioceptive systems. These sensory inputs combine with the appropriate neuromuscular response and flexibilities of joint movements to achieve walking (Smith et al., 2017). However, in daily life, walking is often required to complete other behaviors or perform other thinking activities that are unrelated to walking itself. This ability to simultaneously perform multiple tasks and its impact on attention distribution to each task are the focus of current studies. Dual-task walking is a strong predictor of fall risk, disability, and mortality (Beurskens and Bock, 2012; Holtzer et al., 2017). The extent of the effects of dual tasks on walking depends on factors, such as age, type, and complexity of tasks. Regulation of dual-task walking can improve balancing abilities as well as the ability to selective apply attention (Verghese et al., 2012).

In most sports, higher requirements for coordination and stability between limbs, good limb coordination, and stability are closely associated with the excellent performance of athletes. Therefore, attentional control (AC) seems to be crucial. For instance, professional athletes need a wide breadth of attention, including the position and movement of teammates and opponents in order to perceive unexpected stimuli, thereby generating tactical response patterns and seeking original solutions in the game plan (Memmert and Furley, 2007). In skiing, in addition to skills, diverse external factors, such as temperature, wind, and snow conditions, affect the performance of athletes. Therefore, outcomes depend on how athletes manage their attentional resources, either by concentrating or distributing them (Florina et al., 2014). It has been defined that AC is the ability of an individual to transfer attention from a particular dominant environment to another subordinated information (Derryberry et al., 2003), which reflects the ability of an individual to allocate attention to environmental information (Rothbart et al., 1994) and is also part of cognitive control. Therefore, the cognitive control mechanism in the brain is an executive system that determines how to allocate limited attentional resources, which is crucial for an individual to flexibly and dynamically adjust their performance in response to changing environmental demands and internal goals (Shenhav et al., 2013). Posner et al. reported that AC is associated with functions of the anterior attentional system that is located within the frontal region (anterior cingulate cortex) (Posner and Petersen, 1990; Posner and Rothbart, 1998) and plays a critical role in complex cognitive/attentional processing (Badgaiyan and Posner, 1998; Casey et al., 2015). Since the anterior cingulate cortex is influenced by emotions (Derryberry and Reed, 2002), tasks, and characteristics of individuals (Bush et al., 1999), AC varies with each individual. Enhancement of anterior system functions is associated with stronger voluntary control over orientation, conceptual processing, and behaviors, which allows for greater flexibility and control over dominant tendencies (Derryberry et al., 2003). Individuals with better AC and with a high level of precision and flexibility in controlling behaviors (Derryberry et al., 2003) are better at attenuating their fears, making effective plans and their implementation to be more likely (Compas and Boyer, 2001). Individual differences are associated with diverse ways through which people deal with negative or threatening information, as well as the coping efficiency associated with daily failures (Derryberry and Reed, 2002; Unsworth et al., 2012). Various studies have evaluated the role of AC in sports psychology (Corbetta and Shulman, 2002; Derryberry and Reed, 2002; Derryberry et al., 2003; Eysenck and Derakshan, 2011): however, the impact of AC on motor performance under dual task conditions have not received much attention.

This study aimed at elucidating the impact of cognitive tasks on gait and inter-joint coordination. We determined whether different AC affects gait and coordination in the completion of dual tasks and investigated the relationship between AC and motor control. We hypothesized that compared with High AC (HAC), the Low AC (LAC) group is more susceptible to cognitive tasks, has a weaker ability to maintain gait control and inter-joint coordination, and has a higher risk of falling.

## Methods

### Participants

Fifty young male students were recruited from Soochow University and subjected to the Stroop task. Based on the reaction time (RT) of the Stroop task, they were ranked from low to high. The top 15 participants (30%) were assigned to the HAC group, while the last 15 participants (30%) were assigned to the LAC group (Derryberry and Reed, 2002; Roelofs, 2003) (Table 1). Due to our consideration of gender differences, only men were included in the study. Participants were excluded from the study if they exhibited musculoskeletal pain, have had lower extremity injury during the prior 6 months, exhibited neurological impairments, exhibited cardiovascular or cardiopulmonary problems, and had contraindications to treadmill walking. All the participants were right-handed and heel strike, with a normal or corrected-to-normal vision. Ethical approval was obtained from the Ethics Committee of Soochow University (ECSU), and all the participants provided written informed consent.

**Table 1:** Characteristics of participants per group.

	**LAC (*n* = 15)**	**HAC (*n* = 15)**
Reaction time (ms)	2615.21 (294.11)	1756.31 (127.62)
Age (yr)	21.00 (0.93)	20.67 (1.72)
Height (cm)	178.67 (4.30)	176.53 (7.55)
Weight (kg)	72.16 (8.83)	71.46 (10.36)

*Values are presented as mean (M) (SD) unless stated otherwise*.

### Material and Apparatus

**Figure 1:**
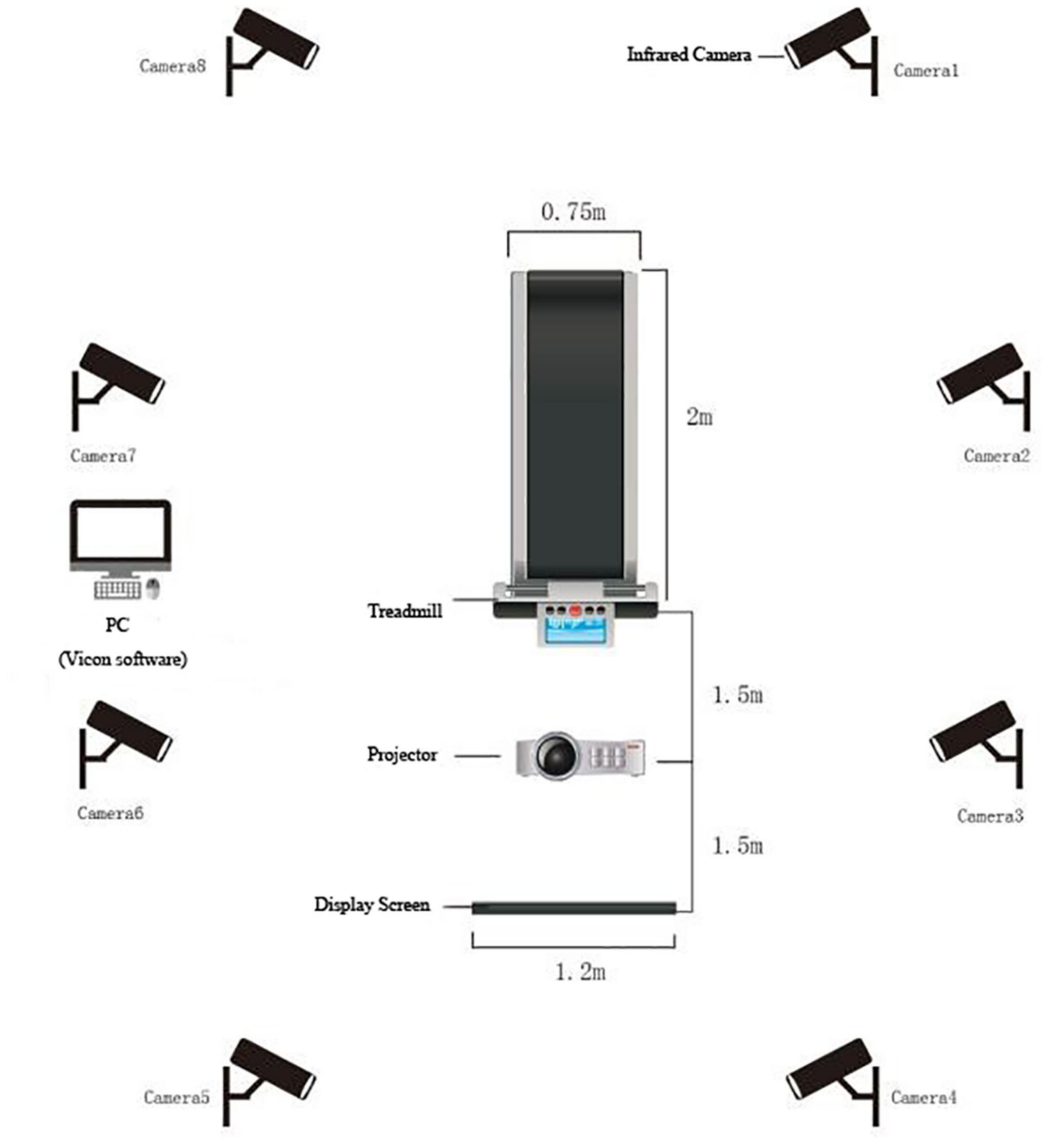
Overview of the experimental setup.

A projector (Panasonic BX30, Panasonic Inc., Osaka, Japan) and a screen placed directly in front of the participants were used to perform cognitive tasks. Response buttons were held in both the left and right hands, and the E-prime software 2.0 (Psychology Software Tools, Inc, Sharpsburg, PA, United States) was used to record RT and correct rate (CR). Gait tests were performed on a motorized treadmill with a large pressure sensor embedded at the speed of 1.33 m/s (ZebrisFDM-T, Zebris Medical GmbH, Isny, Germany), which allowed for online detection of gait characteristics (e.g., gait cycle, gait speed, step length, step speed, step width, step time) (Figure 1). Eight high resolution IR cameras (Vicon, Inc., Oxford, United Kingdom) at a sampling rate of 100 HZ and a Vicon lower body plug-in gait marker set was used to capture kinematic data on the sagittal plane (Davis et al., 1991).

### Experimental Protocol

**Figure 2:**
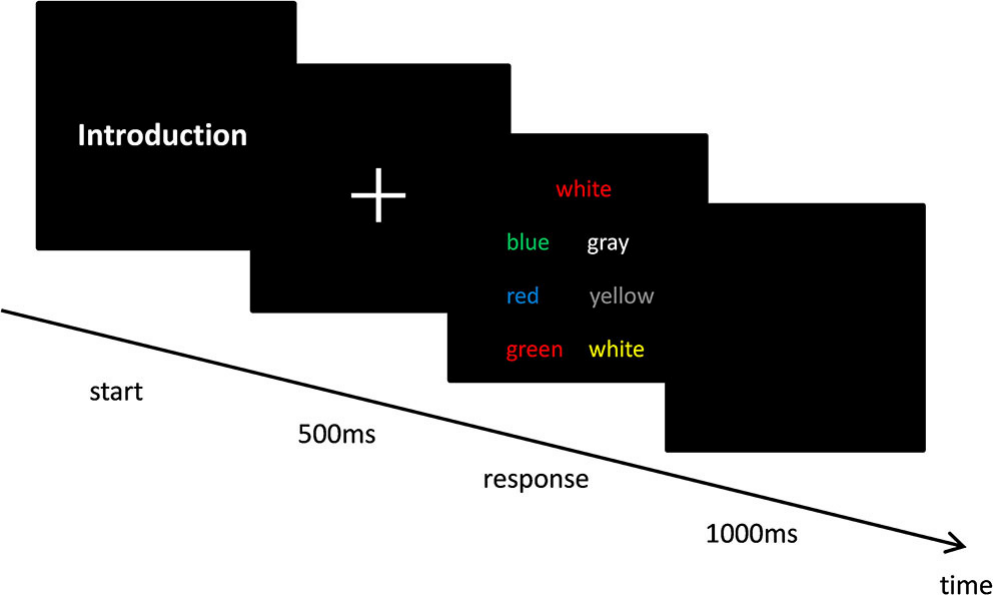
Stroop task experimental process.

To quantitatively measure their ability for AC, 50 young male students were instructed to perform the Stroop task and answer the Attentional Control Scale questionnaire (Derryberry and Reed, 2002). Given the correction (*r* = −0.352, *p* = 0.001) between two measurements, the groups were unequal in AC abilities (Peers and Lawrence, 2009; Judah et al., 2014). In the Stroop task, the participants were asked to select the correct option among six response options as quickly and as accurately as possible while displaying the target and response options on the screen (choose the color that matches the target itself rather than the color of word description). The experiment was repeated 90 times within 10 min and included 10 familiarizations and 80 formal tests. Only after the participants had selected the correct option was the next trial performed. The specific experimental process is as shown in Figure 2.

The experimental protocol consisted of a single task (ST) and a dual task (DT). This study describes gait performance as a motor function and cognitive task performance as a cognitive function. ST contains two conditions: (i) ST cognitive task: 2-back working memory task while sitting; and (ii) ST motor performance: walking. During the ST cognitive task, a series of 25 pseudo-randomized letters (A–J) was consecutively projected on the screen. Each white letter was presented against a black background for 500 ms with an interstimulus interval of 1,900 ms (E-prime2.0). The participants were required to respond after each letter: They pressed a button on the right hand if the letter on the screen matched the letter displayed two stimuli earlier (i.e., two back); otherwise, they pressed a button on the left hand if there was no match. There were 25 letters in the sequence, five of which were correct responses (20% of total stimuli) (Wrightson et al., 2016). Before beginning the ST motor performance test, participants were instructed to perform a familiar trail by walking on the treadmill at a speed of 1.33 m/s. After familiarization, the tests were completed without any cognitive task.

Regarding DT, participants were required to walk at a speed of 1.33 m/s after familiarization while simultaneously performing a 2-back task. They performed three 60-s trials (Potvin-Desrochers et al., 2017) per condition, presented in a randomized order, and counterbalanced across them. They were allowed a 5-min break between each trial (Wollesen et al., 2016) in each condition to prevent fatigue or boredom.

### Data Collection and Analysis

Correct rate and RT as cognitive outcomes were directly acquired by E-prime2.0 during both ST (sitting) and DT (walking). Gait-related parameters were step length, step width, foot progression angle, step time, gait cycle, contact time (forefoot, midfoot, heel), and heel-forefoot time, which were recorded using Zebris during ST (waking) and DT (cognitive-walking). Evaluation of the inter-joint coordination may elucidate essential timing and sequencing of the neuromuscular system control over biomechanical degrees of freedom. Variability of coordination may reflect the adaptability of this control. Moreover, CRP has been used in various studies to identify the pattern and variability of lower limb inter-joint coordination (Burgess-Limerick et al., 1993; Hamill et al., 1999; Lu et al., 2008; Chiu and Chou, 2012; Chiu et al., 2013). In this study, a custom-written MATLAB (Matlab R2013a, MathWorks, Natick, MA, United States) program was used to calculate CRP.

To minimize the influence of different movements in amplitudes and frequencies, normalization was performed to define the values of angular positions (θ) between 1 and −1, with the midpoint located at zero. Angular velocities (ω) were normalized by maximum absolute velocity (Hamill et al., 1999; Li et al., 1999; Chiu and Chou, 2012; Hein et al., 2012) with the following equations:

(1) $$\begin{array}{r} \theta_{i}=\frac{2\times \left[\theta_{i}-\min\left(\theta_{i}\right)\right]}{\max\left(\theta_{i}\right)-\min\left(\theta_{i}\right)}-1 \end{array}$$

(2) $$\begin{array}{r} \omega_{i}=\frac{\omega_{i}}{\max\left\{\left|\omega_{i}\right|\right\}} \end{array}$$

where θ_*i*_ and ω_*i*_represent angular positions and velocity for each data point during a gait cycle. Phase angle (φ) was calculated as $\varphi =\tan^{-1}\left(\frac{\theta }{\omega }\right)$ along each normalized data point and unwrapped to correct discontinuities occurring during angle computation (Chiu and Chou, 2012). Calculated phase angles were in the range of 0–180°, with positive values in the first and second quadrants and negative values in the third and fourth quadrants. Then, four quadrant arctangent phase angles were normalized with the following equations (Hamill et al., 1999):


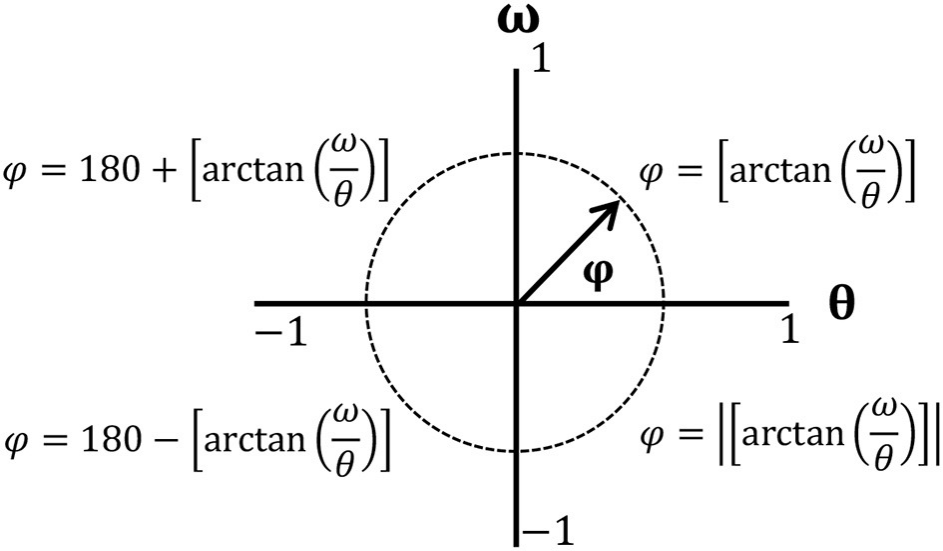


By subtracting the phase angle of the distal joint from that of the proximal joint (φ_*Hip*−*Knee*_ = φ_*Hip*_ − φ_*Knee*_;φ_*Knee*−*Ankle*_ = φ_*Knee*_ − φ_*Ankle*_), relative phase angles (RPAs) were obtained to identify adjacent joint coordination (hip-knee or knee-ankle) (Burgess-Limerick et al., 1993). If an RPA is close to 0° or ± 360°, adjacent joints move in a similar fashion or in-phase. If an RPA approaches ±180°, the adjacent joints move in an opposite fashion or out-of-phase (Hamill et al., 1999; Stergiou et al., 2001).

Differences in all points of the ensemble CRP curve over the stance and swing phases of a gait cycle were examined with mean absolute relative phase (MARP) and deviation phase (DP) (Stergiou et al., 2001). The MARP over stance and swing phases of a gait cycle was calculated to evaluate phase relations between joints.

A MARP value that is close to 0 indicates synchronous oscillation between the joints (Stergiou et al., 2001). The DP represented trial-to-trail variability and was used to compare systemic inter-joint characteristics within the stance and swing phases of a gait cycle. A high DP value indicated high coordination variability between two joints (Hamill et al., 1999; Stergiou et al., 2001).

### Statistical Analysis

Mean (M) and SD of the parameters were calculated using a two-way repeated measures ANOVA with group as the between-subject factor (HAC, LAC) and task as the within-subject factor (ST, DT). Paired sample *t*-tests were performed to test differences between ST and DT in LAC and HAC, respectively. Significance was set at *p* < 0.05. All statistical analyses were performed using the software SPSS statistics (17.0, IBM Inc., Chicago, IL, United States).

## Results

### Cognitive Task Outcomes

**Figure 3:**
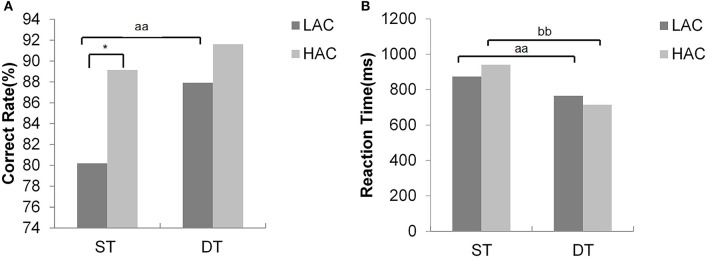
**(A)** Value of correct rate (%) and **(B)** value of reaction time. *significant group difference; ^aa^significant task main effects between single task (ST) and dual task (DT) for low attentional control (LAC); ^bb^significant task main effects between ST and DT for high attentional control (HAC).

The mean CR of HAC was higher than that of LAC in ST. Moreover, LAC exhibited significant differences in RT and CR (*t* = 4.08, *p* = 0.001; *t* = −3.57, *p* = 0.003). However, HAC only exhibited significant differences in RT (*t* = 8.02, *p* = 0) (Table 2, Figure 3).

**Table 2:** M (SD) values of cognitive outcomes during ST and DT for LAC and HAC.

	**LAC**		**HAC**		**Group difference**	**Task difference**
	**ST**	**DT**	**ST**	**DT**	***F* and *P*-value**	***F* and *P*-value**
CR (%)	80.19 (11.22)^*^^aa^	87.92 (7.52)^aa^	89.13 (4.80)^*^	91.61 (4.03)	***F*** **= 7.00**, ***p*** **= 0.013**	***F*** **= 15.44**, ***p*** **= 0.001**
RT (ms)	874.78 (163.11)^aa^	765.40 (194.17)^aa^	940.32 (200.51)^bb^	714.13 (207.16)^bb^	*F* = 0.01, *p* = 0.916	***F*** **= 74.31**, ***p*** **= 0.001**

*ST, single task; DT, dual task; LAC, low attentional control; HAC, high attentional control; CR, correct rate; RT, reaction time*.

* *Represents a significant effect*.

* *indicates p < 0.05;*

aa *indicates p < 0.01;*

bb *indicates p < 0.01. Bold values: significant p-values*.

### Gait-Related Parameters

There were significant task effects on step width (t = 2.69, *p* = 0.018), support phase (*t* = 2.67, *p* = 0.018; *t* = 4.32, *p* = 0.001, respectively), swing phase (*t* = −2.67, *p* = 0.018; *t* = −4.32, *p* = 0.001, respectively), double-support phase (*t* = 3.6, *p* = 0.003), right step time (*t* = −2.48, *p* = 0.027), left forefoot and right forefoot contact time (*t* = 4.46, *p* = 0.001; *t* = 3.85, *p* = 0.002, respectively), and left heel-forefoot time in LAC (*t* = −2.96, *p* = 0.01). However, significant task effects were only found on left foot progression angle (*t* = −2.23, *p* = 0.043).

Comparisons of gait-related parameters between LAC and HAC revealed that LAC was performed with longer heel contact times of left foot and right foot and longer heel-forefoot times of left foot and right foot in both conditions. None of the other gait-related parameters differed significantly between the groups (*p* > 0.05). Spatiotemporal gait parameters under the two conditions are shown in Table 3.

**Table 3:** M (SD) value of spatiotemporal gait parameters during ST and DT for LAC and HAC.

		**LAC**		**HAC**		**Group difference**	**Task difference**
		**ST**	**DT**	**ST**	**DT**	***F* and *P*-value**	***F* and *P*-value**
Foot progression angle (°)	L	5.49 (5.12)	5.88 (5.24)	7.52 (4.20)^b^	8.48 (4.89)^b^	*F* = 1.74, *p* = 0.198	***F*** **= 5.138**, ***p*** **= 0.031**
	R	8.84 (4.74)	9.56 (5.10)	9.25 (3.34)	10.10 (4.40)	*F* = 0.09, *p* = 0.768	*F* = 3.83, *p* = 0.060
Step length (/height)		0.75 (0.04)	0.76 (0.02)	0.76 (0.03)	0.77 (0.03)	*F* = 1.30, *p* = 0.263	***F*** **= 5.11**, ***p*** **= 0.032**
Step width (cm)		12.93 (3.92)^a^	11.69 (2.95)^a^	10.67 (2.53)	10.20 (2.83)	*F* = 2.96, *p* = 0.096	***F*** **= 7.93**, ***p*** **= 0.009**
Support phase (%)	L	62.29 (1.12)^a^	61.36 (1.93)^a^	62.00 (1.16)	61.71 (1.48)	*F* = 0.01, *p* = 0.944	***F*** **= 7.45**, ***p*** **= 0.011**
	R	62.82 (1.20)^aa^	61.73 (1.71)^aa^	62.49 (1.04)	62.10 (1.02)	*F* = 0.00, *p* = 0.958	***F*** **= 14.77**, ***p*** **= 0.001**
Swing phase (%)	L	37.71 (1.12)^a^	38.64 (1.93)^a^	38.00 (1.16)	38.29 (1.48)	*F* = 0.01, *p* = 0.944	***F*** **= 7.45**, ***p*** **= 0.011**
	R	37.18 (1.20)^aa^	38.2 (1.71)^aa^	37.51 (1.04)	37.90 (1.02)	*F* = 0.00, *p* = 0.958	***F*** **= 14.77**, ***p*** **= 0.001**
Double-support phase (%)		25.10 (2.18)^aa^	23.09 (3.59)^aa^	24.47 (1.83)	23.81 (2.18)	*F* = 0.00, *p* = 0.957	***F*** **= 12.81**, ***p*** **= 0.001**
Step time (s)	L	0.49 (0.02)	0.50 (0.01)	0.50 (0.03)	0.50 (0.03)	*F* = 0.09, *p* = 0.766	*F* = 4.14, *p* = 0.052
	R	0.48 (0.02)^aa^	0.51 (0.01)^aa^	0.49 (0.03)	0.50 (0.03)	*F* = 0.28, *p* = 0.599	***F*** **= 8.78**, ***p*** **= 0.006**
Forefoot contact time (%)	L	90.86 (1.80)^aa^	89.32 (1.84)^aa^	90.96 (2.24)	90.66 (2.72)	*F* = 0.91, *p* = 0.348	***F*** **= 12.26**, ***p*** **= 0.002**
	R	90.73 (1.53)^aa^	89.35 (1.93)^aa^	91.15 (2.38)	90.50 (2.87)	*F* = 1.03, *p* = 0.320	***F*** **= 15.52**, ***p*** **= 0.001**
Midfoot contact time (%)	L	71.23 (4.90)	70.68 (5.27)	70.65 (4.61)	71.33 (5.38)	*F* = 0.00, *p* = 0.986	*F* = 0.01, *p* = 0.918
	R	70.31 (5.16)	70.16 (5.52)	71.15 (4.07)	69.98 (6.28)	*F* = 0.03, *p* = 0.858	*F* = 0.99, *p* = 0.33
Heel contact time (%)	L	50.09 (7.29)^*^	51.43 (7.73)^*^	44.75 (6.64)^*^	44.89 (7.72)^*^	***F*** **= 5.34**, ***p*** **= 0.028**	*F* = 0.90, *p* = 0.351
	R	49.95 (6.90)^*^	50.60 (7.28)^*^	42.88 (7.03)^*^	44.04 (8.86)^*^	***F*** **= 7.33**, ***p*** **= 0.01**	*F* = 0.64, *p* = 0.429
Heel-forefoot time (%)	L	31.98 (5.51)^a^^*^	34.39 (5.19)^a^^*^	27.77 (7.23)^*^	29.92 (4.36)^*^	***F*** **= 5.44**, ***p*** **= 0.027**	***F*** **= 6.31**, ***p*** **= 0.018**
	R	31.35 (5.76)^*^	33.59 (5.05)^*^	26.37 (8.02)^b^^*^	29.96 (4.83)^b^^*^	***F*** **= 5.65**, ***p*** **= 0.025**	***F*** **= 5.33**, ***p*** **= 0.029**

*ST, single task; DT, dual task; LAC, low attentional control; HAC, high attentional control; CR, correct rate; RT, reaction time*.

*Represents a significant group effect. ^a^indicates a significant effect for LAC group. ^b^indicates a significant effect for HAC group.

* *indicates p < 0.05;*

a and aa *indicate p < 0.05, p < 0.01, respectively;*

b *indicates p < 0.05. Bold values: significant p-values*.

### Inter-joint Coordination

**Figure 4:**
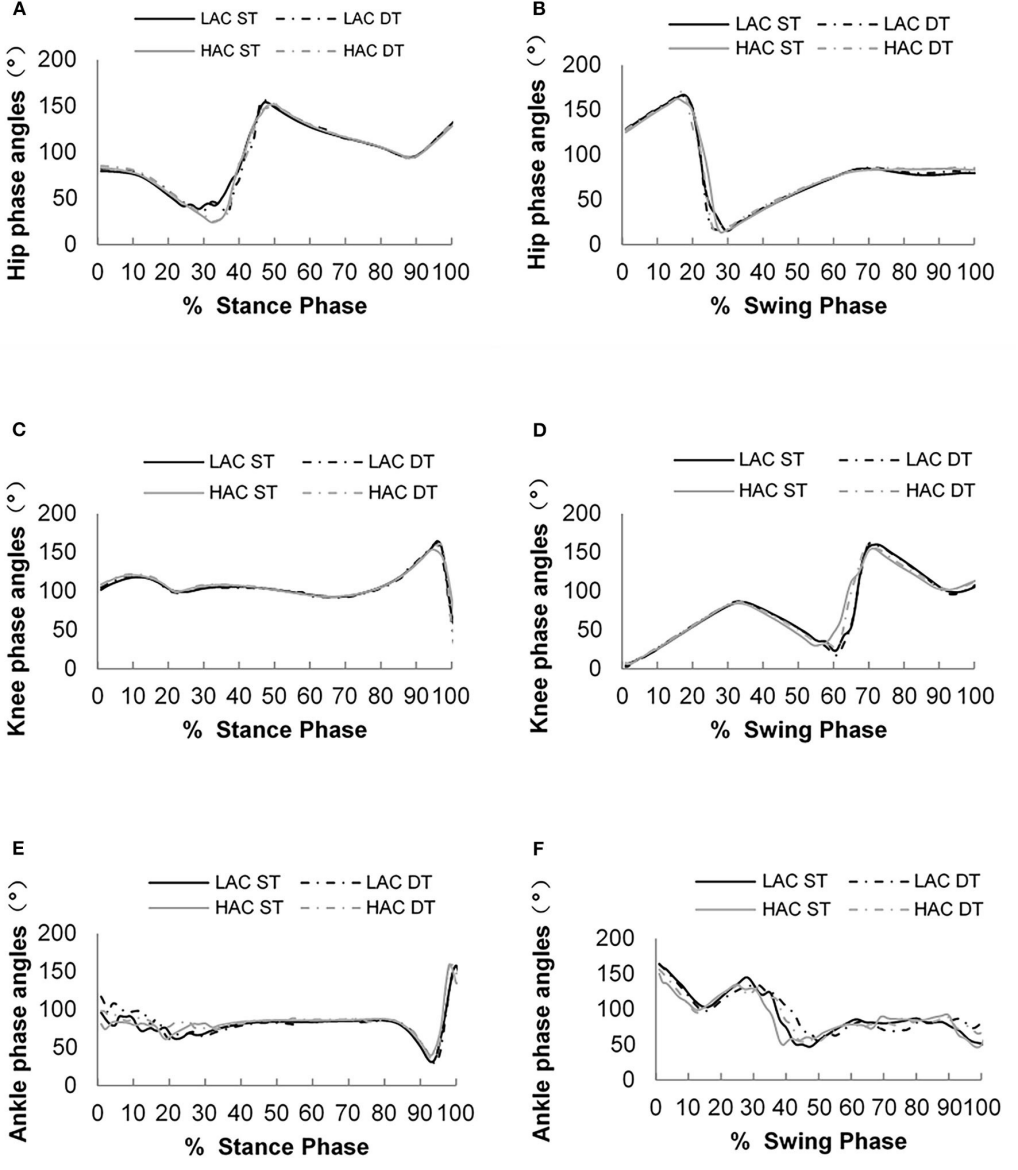
**(A)** Phase angles of the hip joint during the stance phase; **(B)** phase angles of the hip joint during the swing phase; **(C)** phase angles of the knee joint during the stance phase; **(D)** phase angles of the knee joint during the swing phase; **(E)** phase angles of the ankle joint during the stance phase; and **(F)** phase angles of the ankle joint during the swing phase.

**Figure 5:**
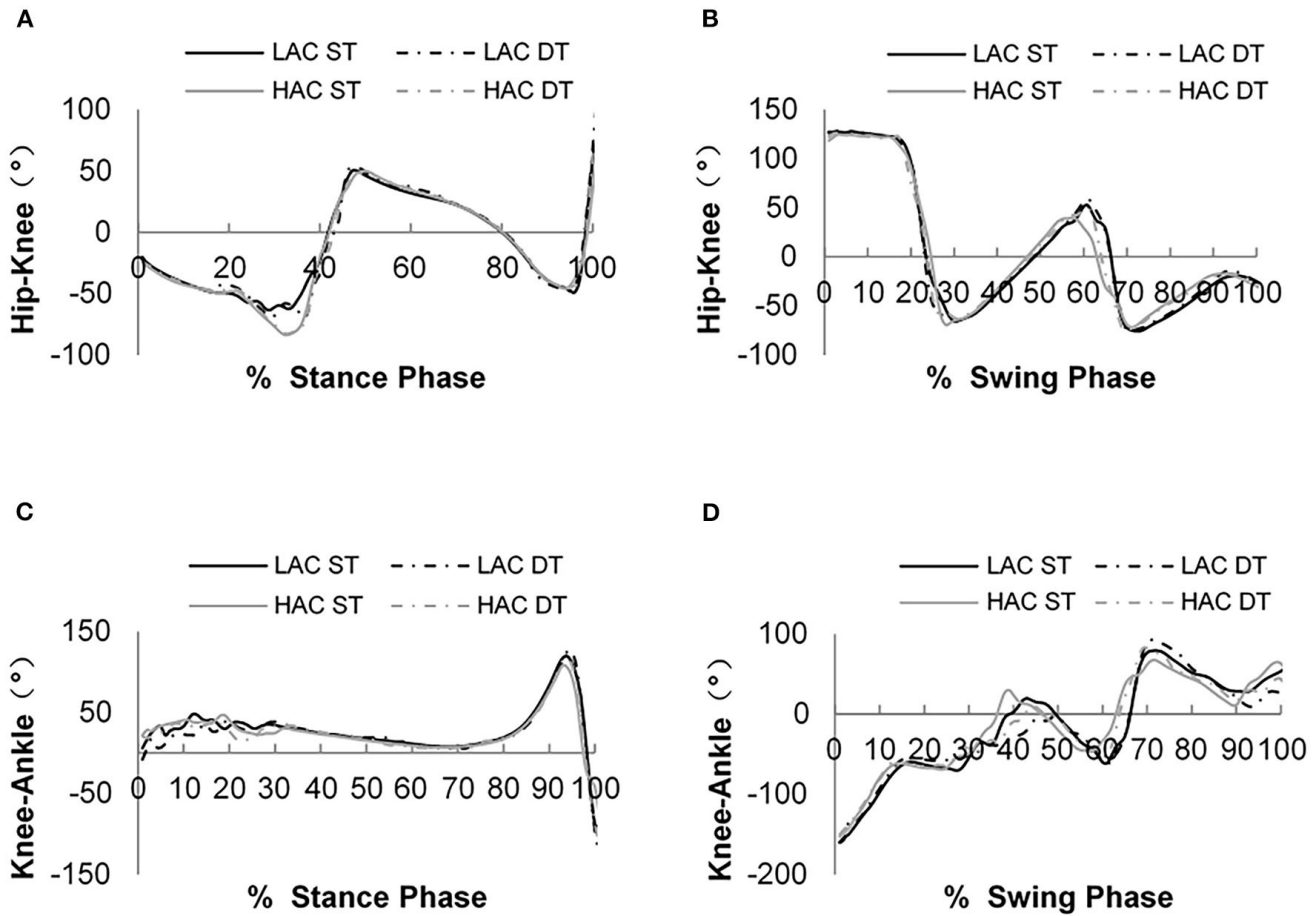
**(A)** Hip–knee continuous relative phase (CRP) curves during the stance phase; **(B)** hip–knee CRP curves during the swing phase; **(C)** hip-knee CRP curves during the stance phase; and **(D)** knee–ankle CRP curves during the swing phase.

Figures 4, 5 show phase angles of the hip, knee, and ankle joints of LAC and HAC under the two walking conditions and mean hip-knee and knee-ankle CRP curves of LAC and HAC under the two walking conditions, respectively.

**Figure 6:**
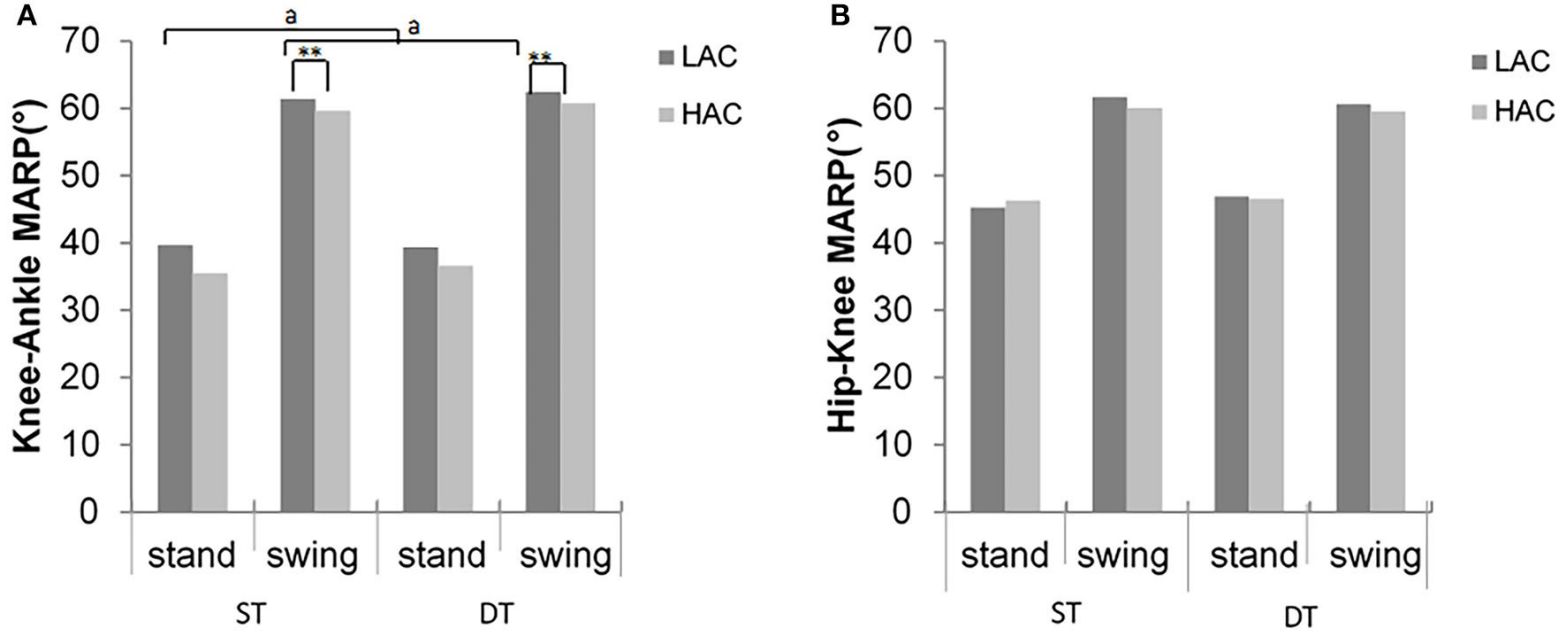
Mean absolute relative phase (MARP) values of **(A)** hip–knee and **(B)** knee–ankle inter-joint coordination for LAC and HAC groups in stance and swing phases during ST and DT (**significant group difference between LAC and HAC at 0.01; ^a^significant task main effects between ST and DT for LAC at 0.05).

**Figure 7:**
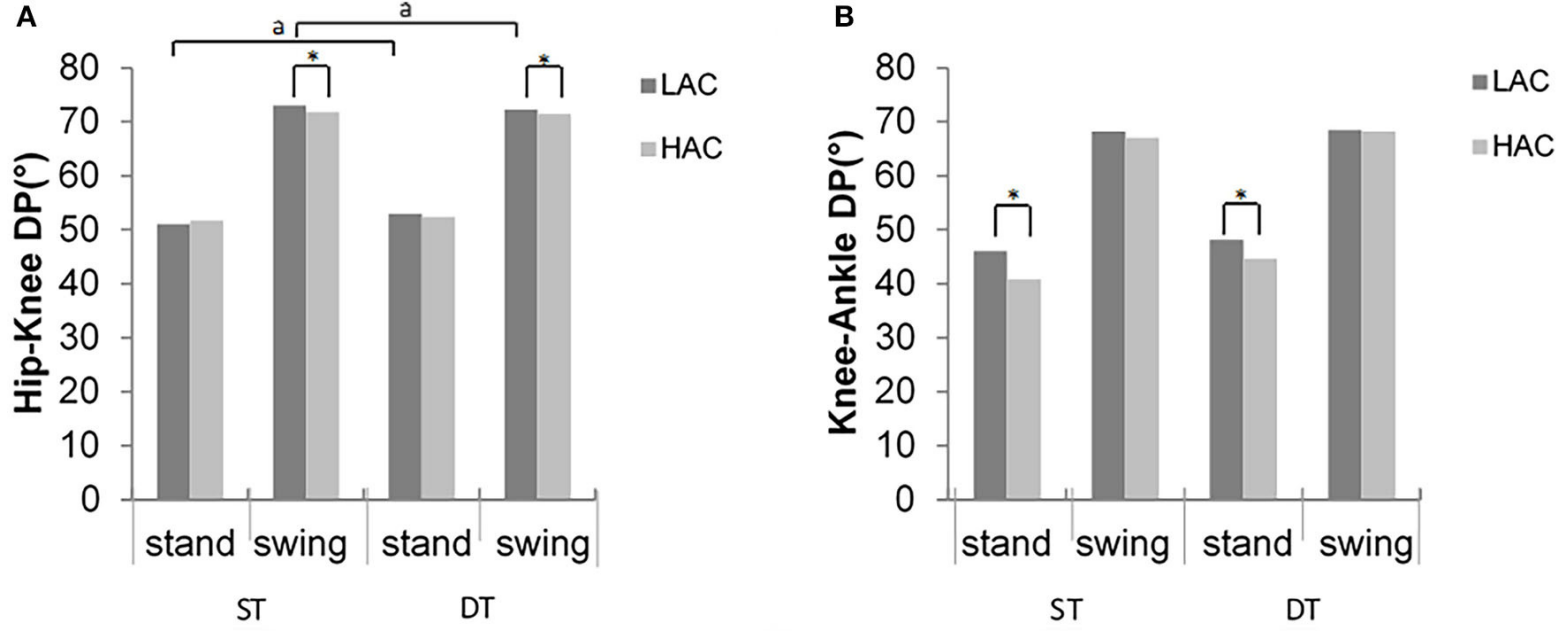
Deviation phase (DP) values of **(A)** hip–knee and **(B)** knee–ankle inter-joint coordination for LAC and HAC groups in stance and swing phases during ST and DT (*significant group difference; ^a^significant task main effects between ST and DT for LAC).

Significant differences between gait conditions were detected in hip-knee MARP and DP in the stance phase for LAC (*t* = −2.89, *p* = 0.012; *t* = −2.5, *p* = 0.026, respectively), consistent with the swing phase (*t* = 2.82, *p* = 0.014; *t* = −2.3, *p* = 0.038, respectively). However, there were no statistically significant differences in HAC (*p* > 0.05) (Figures 6, 7).

In the swing phase, HAC demonstrated significantly smaller (closer to zero) MARP and weaker hip-knee DP values in the swing phase across all gait conditions compared with LAC (*F* = 4.9, *p* = 0.35; *F* = 4.2, *p* = 0.05, respectively, Figure 6). Similarly, during the stance phase, HAC exhibited smaller MARP (closer to 0) values when compared with LAC (*F* = 4.74, *p* = 0.038, respectively, Figure 7).

## Discussion

In this study, we evaluated whether cognitive and motor performances under dual task conditions are different with different AC levels. As expected, CR was higher for HAC than for LAC in ST, which shed light on different levels of AC between the two groups (Derryberry and Reed, 2002; Derryberry et al., 2003). There were significant changes in CR, RT, gait cycle, and lower limb coordination for LAC, while there were no changes in HAC, except for progression angle of the left foot. These outcomes indicate that different ACs are associated with different sensitivities to cognitive interference, consistent with previous findings that the ability to allocate attention resources vary with AC (Derryberry and Reed, 2002; Derryberry et al., 2003; Lonigan and Vasey, 2009). It has been reported that in LAC, with a higher sensitivity to distractions, it might be more difficult to ignore threatening distractors, even if these are known to be task-irrelevant (Paulewicz et al., 2012). However, it might be easier for HAC, with a high level of precision and flexibility in controlling behavior (Derryberry et al., 2003) and attention, to intentionally change the way in which their attention reacts to the presence of certain task-irrelevant stimuli (Derryberry and Reed, 2002). Hence, compared with HAC, it can be inferred that LAC may allocate more attention to cognitive tasks and less attention to walking or other movements.

Previous studies on ST have reported slower gait velocity, longer support time and step time, shorter swing time, narrower step width, shorter step length, and greater inter-joint coordination variability than those of DT (Plummer-D'Amato et al., 2008; Nordin et al., 2010; Peper et al., 2012; Chiu et al., 2013). In this study, LAC exhibited a shorter support time and forefoot contact time but a longer swing time, step time, and heel-forefoot time in DT. These unexpected results should be considered in the type of cognitive task. According to the U-shaped non-linear interaction model, the additional task, with varying cognitive challenges, may either improve or diminish balance performance (Huxhold et al., 2006). Nordin E et al. reported that gait control is attention demanding; however, not all cognitive tasks affect gait in the same manner (Nordin et al., 2010). Moreover, pattern changes in hip–knee inter-joint coordination in the stance phase induced by cognitive task in LAC were greater than those in ST, while the change in the swing phase was weaker than that in ST. Since walking requires adequate integration of peripheral information and communication between spinal and supraspinal structures (Fukuyama et al., 1997), peripheral information from somatosensory systems plays different roles in gait regulation that can be dependent on different gait phase. Therefore, dynamic postural demands and attentional requirements during walking vary from one phase to another (Regnaux et al., 2008; Abbud et al., 2009; Plummer-D'amato et al., 2010; Lo et al., 2017), resulting in different inter-joint coordination according to the phase of the gait cycle. Another explanation may be found in longer swing time for adjusting the posture and shorter stance time for adjusting the posture in DT.

Significant differences were found in contact times of heel, heel–forefoot times, and inter-joint coordination between LAC and HAC. First, LAC exhibited longer contact time of heel and heel–forefoot time, which indicated that when walking, LAC moved slower than HAC. Second, we found that changes in hip–knee inter-joint coordination patterns during the swing phase and knee–ankle inter-joint coordination patterns during the stance phase of LAC were greater than those of HAC. These findings imply that HAC exhibits a more stable gait, with better hip–knee coordination patterns during the swing phase and knee–ankle coordination patterns during the stance phase. Since, coordination is important in maintaining dynamic balance (Winter, 1992; Lacquaniti et al., 1997), more out-of-phase coordination and greater hip–knee and knee–ankle inter-joint coordination variability in LAC compared with HAC could be contributing factors to gait imbalance in LAC (Chiu et al., 2013). Visual information plays a significant role in overcoming obstacles during the swing phase (Mcfadyen et al., 2007), and that visual inputs are vital in determining whether a participant can accurately place the foot on the ground in the terminal swing (Bent et al., 2004). Furthermore, the CNS selects an efficient control strategy that exploits lower limb dynamics to accomplish visual disturbance conditions, especially for controlling proximal joints (hip and knee) (Mcfadyen and Carnahan, 1997; Chiu et al., 2013). The hip, the most proximal joint of the lower limb, offered a more efficient means of elevating the swing toe than the knee and ankle (Lu et al., 2006). In this study, greater hip–knee coordination variability during the swing phase in LAC was associated with elevating the whole limb with increased knee flexion rather than the hip, which may be responsible for greater foot obstacle clearance and higher tripping incidents during the swing phase for LAC. Proximal joints play a greater role in balance control (Winter, 1995; Chiu and Chou, 2012), but the adjustment of distal joints might also be essential for accommodating complex walking tasks (Chiu et al., 2013). For example, in the stance phase, the ankle strategy was used in maintaining balance as a single-segment inverted pendulum by generating torque at the ankle during quiet standing (Karlsson and Lanshammar, 1997; Colobert et al., 2006; Lu et al., 2008; Gatev et al., 2010). To maintain stability during walking, humans normally do not significantly adjust the sagittal plane position of the foot but rather adjust the center of pressure position and increase the push-off force to reduce the effort associated with maintaining stability during walking, reflecting the use of ankle joint moment (Bruijn and van Dieën, 2018). Therefore, decreased stability of knee–ankle coordination in the stance and swing phases may indicate increased difficulty in ankle joint control when modulating the stability of the body during LAC.

Therefore, differences in attention have a certain impact on movement stability and joint coordination, implying that we may need to select the population with HAC for sports with high requirements on movement coordination and movement stability. However, it has not been established whether it is possible to enhance athletic performance by training AC. This study is associated with some limitations. It has been established that we use visual targets (Rushton et al., 1998) to navigate through our environment and need specific topographic information to secure adequate foot placement. However, this study involved walking on the treadmill while looking ahead, where space was confined and fixed and unlike over-ground. Therefore, in both ST and DT, the participants could have partly distracted their attention resources when walking, which explains the same significant differences between LAC and HAC regardless of ST or DT. Another limitation is that we did not assess how the other types of DT interfered with different cognitive tasks. We only evaluated one type of DT, and further studies are needed to assess and validate gait and inter-joint coordination with different types of DT. In addition to target action performance, sports performance includes various interlaced connections between psychology and physiology. We only evaluated the performance of walking movement, which has some limitations. More studies are needed to evaluate the influence of AC on sports performance from other perspectives.

## Conclusion

In conclusion, LAC devotes fewer attention resources to the regulation of walking, resulting in poor gait adjustment ability and greater inter-joint coordination variability to perturbations. This finding implies that AC influences the ability to maintain gait control and modulate inter-joint coordination patterns when accommodating gait perturbations. Such differences may be explained, in part, by LAC limitations in cognitive flexibility, which is correlated with the reallocation of CNS capacity or resources. The findings suggest that there may be a correlation between AC ability and sports performance, which may be used to provide theoretical support for future studies on athlete selection and reduction of fall risk in a specific population.

## Data Availability Statement

The original contributions presented in the study are included in the article/Supplementary Material, further inquiries can be directed to the corresponding author/s.

## Ethics Statement

The studies involving human participants were reviewed and approved by Ethics Committee of Soochow University (ECSU). The patients/participants provided their written informed consent to participate in this study.

## Author Contributions

CW, GW, and YZ prepared the original manuscript, figures, and tables. CW and GW analyzed the data, interpreted the results, and drafted the document. AL participated in the elaboration of experimental design and data interpretation. All authors approved the submitted version and contributed to the article.

## Conflict of Interest

The authors declare that the research was conducted in the absence of any commercial or financial relationships that could be construed as a potential conflict of interest.
