# Supplementary material for: Effects of Attentional Control on Gait and Inter-Joint Coordination During Dual-Task Walking
Source: Front Psychol. 2021 Jul 21;12:665175. doi: 10.3389/fpsyg.2021.665175 (PMC8334006; doi:10.3389/fpsyg.2021.665175)
Supplement: Supplementary file 1 [file Data_Sheet_1.docx]

**注意控制量表（Attentional control scale， ACS）**

姓名 出生年月日 联系方式

1. 当周围有噪音时，我很难集中精力去做一件困难的事情。

€ 总是 € 经常 € 有时候 € 几乎不

1. 当我需要集中精力解决问题时，我无法集中注意力。

€ 总是 € 经常 € 有时候 € 几乎不

1. 当我正在努力工作时，我会被周围的事情分心。

€ 总是 € 经常 € 有时候 € 几乎不

1. 即使在房间里放音乐，我也能集中注意力。

€ 总是 € 经常 € 有时候 € 几乎不

1. 当我集中注意力的时候，我不能注意到房间里发生的其他事情。

€ 总是 € 经常 € 有时候 € 几乎不

1. 当我再读书或学习时，如果房间里有人说话，我很容易分心。

€ 总是 € 经常 € 有时候 € 几乎不

1. 当我想把注意力集中在某事上时，很难阻止分散注意力想法的出现。

€ 总是 € 经常 € 有时候 € 几乎不

1. 当我对某事感到兴奋时，我很难集中注意力。

€ 总是 € 经常 € 有时候 € 几乎不

1. 当我集中注意力时，我很难忽略饥饿或口渴的感觉。

€ 总是 € 经常 € 有时候 € 几乎不

1. 我可以快速从一个任务转换到另一个任务。

€ 总是 € 经常 € 有时候 € 几乎不

1. 我需要花一些时间才能真正参与到新的任务中。

€ 总是 € 经常 € 有时候 € 几乎不

1. 对我来说，听课记笔记时所需要的听和写动作很难协调在一起。

€ 总是 € 经常 € 有时候 € 几乎不

1. 当我需要的时候，我可以很快地对一个新话题产生兴趣。

€ 总是 € 经常 € 有时候 € 几乎不

1. 当我正在打电话时，我可以轻易读写或写东西。

€ 总是 € 经常 € 有时候 € 几乎不

1. 我很难同时进行两个对话。

€ 总是 € 经常 € 有时候 € 几乎不

1. 我很难快速提出新想法。

€ 总是 € 经常 € 有时候 € 几乎不

1. 在被打扰或分心之后，我可以很容易地将注意力重新转移到我之前做的事情上。

€ 总是 € 经常 € 有时候 € 几乎不

1. 当脑海中有一个分心的想法，我可以轻易克服这个想法的干扰。

€ 总是 € 经常 € 有时候 € 几乎不

1. 我可以轻易地在两个不同任务之间转换。

€ 总是 € 经常 € 有时候 € 几乎不

1. 我很难摆脱一种思维模式，产生另一种新的思维方式。

€ 总是 € 经常 € 有时候 € 几乎不

**Attentional Control Scale**

Name: Date of Birth: Phone Number:

Items are scored on a 4-point scale (1 almost never; 2 sometimes; 3 often; 4 always). R reverse-scored item.

1. It’s very hard for me to concentrate on a difficult task when there are noises around. (R)
2. When I need to concentrate and solve a problem, I have trouble focusing my attention. (R)
3. When I am working hard on something, I still get distracted by events around me. (R)
4. My concentration is good even if there is music in the room around me.
5. When concentrating, I can focus my attention so that I become unaware of what’s going on in the room around me.
6. When I am reading or studying, I am easily distracted if there are people talking in the same room. (R)
7. When trying to focus my attention on something, I have difficulty blocking out distracting thoughts. (R)
8. I have a hard time concentrating when I’m excited about something. (R)
9. When concentrating I ignore feelings of hunger or thirst.
10. I can quickly switch from one task to another.
11. It takes me a while to get really involved in a new task. (R)
12. It is difficult for me to coordinate my attention between the listening and writing required when taking notes during lectures. (R)
13. I can become interested in a new topic very quickly when I need to.
14. It is easy for me to read or write while I’m also talking on the phone.
15. I have trouble carrying on two conversations at once. (R)
16. I have a hard time coming up with new ideas quickly. (R)
17. After being interrupted or distracted, I can easily shift my attention back to what I was doing before.
18. When a distracting thought comes to mind, it is easy for me to shift my attention away from it.
19. It is easy for me to alternate between two different tasks.
20. It is hard for me to break from one way of thinking about something and look at it from another point of view. (R)
