# Supplementary material for: Effects of Attentional Control on Gait and Inter-Joint Coordination During Dual-Task Walking
Source: Front Psychol. 2021 Jul 21;12:665175. doi: 10.3389/fpsyg.2021.665175 (PMC8334006; doi:10.3389/fpsyg.2021.665175)
Supplement: Supplementary file 2 [file Data_Sheet_2.DOCX]

**知情同意书Informed Consent**

| 姓名  Name |  | | 性别  Gender |  | 出生日期  Date of Birth |  |
| --- | --- | --- | --- | --- | --- | --- |
| 身高  Height | cm | | 体重  Weight | kg | 专业  Major |  |
| 联系电话  Phone Number |  | | | 运动强度  Exercise Intensity | |  |
| 一周运动次数Number of exercises per week | |  | | 运动项目  Sports Events | |  |

| 协议题目  Agreement subject |  | |
| --- | --- | --- |
| 研究者  investigator |  | |
| 合作研究者  Co-investigator |  |  |
| 被试类型  The participants type | 志愿者volunteer | |

**实验目的/背景信息：**

感谢您参与此项目，此项目是关于“认知任务干扰对不同注意控制人群步态的影响”，它将历时2小时，由于您符合被试的条件，特邀您参加此项实验。

针对步行这个日常动作，通常会受到额外认知任务干扰从而造成注意力分散，直接影响功能性动作控制和感觉系统信息整合能力，因此我们针对此问题设计本实验。实验目的是通过注意控制能力测试（stroop任务）筛选出高注意控制能力组和低注意控制能力组，比较不同注意控制能力人群在认知任务干扰下步行动作变化的差异性.

**测试流程：**

在实验前根据注意控制能力测试，将平均反应时从低到高排列，将前30%的参与者分配为高注意控制能力组，后30%的参与者分配为低注意控制能立组，然后将选取的受试者进行动作测试（具体测试内容见实验流程图）。

**安全性：**

本实验采取的实验仪器：vicon红外运动捕捉系统和8台MX13型号摄像头捕捉运动学数据，采样频率为100HZ，软件版本为viconnexus 1.5.1，依据人体解剖标志及系统三维重构的基本要求，分别将（16）个直径为14mm的marker球准确置放于人体各环节的标志点上。采用意大利生产的Cometa Wave Wireless表面肌电系统 ，采样频率为2000HZ，对原始肌电信号进行整流和滤波（10-500HZ）处理。采用德国生产的Zebris步态分析跑台，对受试者在步行时的步态运动学参数进行采集。这些仪器均为可靠的测试器械，测试时不需要做特殊的准备（无须注射或服用任何东西），也不用抽血。

我们预计本次实验是没有任何明显不良影响的。但是如果您有强烈的反应，您必须告知实验人员。

**终止权：**

您可以在任何时候退出实验，并且可以不透露您的退出理由。

**保密性：**

关于您的姓名以及您在本次实验的情况都会得到保密，您提供的信息和数据仅供内部的研究分析，不会用于任何的广告宣传（如果我们有必要把您的个人情况用于其它方面，我们将会进一步与您达成一致。）。所有的相关资料，我们会保存三年，并在三年后销毁。

同时，我们希望您认真对待实验，听从测试者的实验安排并积极配合，建议您对实验期间得到的所有信息保密。

**联系人：**

如果您对于此次研究实验有任何的疑问，请联系以下的主试人员：

| 姓名：XXX 电话：XXX  Email：XXX | 姓名：XXX 电话：XXX  Email：XXX |
| --- | --- |

志愿者受试声明：

我做出承诺和保证如下：

1. 我已经仔细阅读了上述项目研究的详细资料，并了解实验流程。
2. 我真实回答了主试者提出的所有有关实验的相关问题。
3. 我知道工作人员在正式实验前对我进行评估，以确保我是否有资格参加这项研究。
4. 我同意遵守实验要求，积极配合实验，坚持按时保质保量完成所有实验项目。在实验过程中，一旦发生意外或异常情况，我会马上报告。
5. 我清楚在与实验相关的报告及论文等相关文件中不会出现自己的姓名，在有保密措施的前提下，我同意以任何方式使用研究成果。
6. 我会对实验期间得到的所有信息保密。

被试（志愿者）

我声明我已经被告知本实验的目的和过程。我的所有问题都得到了满意回答，我下面的签名表明我愿意参加本次研究。

签名________________

主试（研究者）

我已经解释了研究的目的、流程、潜在危险以及被试的权益，并最大可能回答了与研究有关的问题。

签名________________

日期________________

**Experimental Objective/Background Information：**

Thank you for your participation in this project, which is about the influence of cognitive task interference on the gait of different attention-control groups. It will last 2 hours. Since you meet the conditions of the subject, we sincerely invite you to participate in this experiment.

For the daily movement of walking, attention is usually distracted by additional cognitive tasks, which directly affects the functional motor control and information integration ability of the sensory system. Therefore, we designed this experiment to explore this question. The purpose of the experiment was to screen out the high attentional control group and the low attentional control group through the Stroop task, and to compare the differences in the changes of walking movements of people with different attention-control abilities under the interference of cognitive tasks.

**Testing Process:**

Before the experiment, the average reaction time was ranked from low to high according to the test of attention control ability. The first 30% of participants were assigned to the group with high attention control ability, while the last 30% were assigned to the group with low attention control ability. Then the selected subjects were tested for relevant actions.

Safety:

The experimental instruments adopted in this experiment include: Vicon infrared motion capture system and 8 cameras (MX13) to capture kinematic data with a sampling frequency of 100HZ. The software version is ViconNexus 1.5.1. According to the basic requirements of human anatomical markers and three-dimensional reconstruction of the system, (16) marker balls with a diameter of 14mm were accurately placed on the marker points of each link of the human body. Cometa Wave Wireless surface EMG system（Italy）was adopted. The sampling frequency was 2000HZ, and the original EMG signals were rectified and filtered (10-500Hz). Zebris gait analysis platform (Germany) was adopted to collect kinematic parameters of subjects' gait during walking. These are reliable test instruments that require no special preparation (no injections or pills) or blood drawing.

We do not expect any obvious adverse effects from this experiment, but if you have a strong reaction, you must inform the experimenter immediately.

**Right of Termination:**

You may withdraw from the experiment at any time, and you may not disclose your reasons for withdrawal.

[**Privacy**](javascript:;)**:**

The information and data provided by you will only be used for internal research and analysis and will not be used for advertising purposes. (If it is necessary for us to use your personal information for other purposes, we will further agree with you.)

Meanwhile, we hope that you take the experiment seriously, follow the experimenter's arrangement and actively cooperate with us. We suggest that you keep all the information obtained during the experiment confidential.

**Contacts:**

If you have any questions about this study, please contact the investigator below：

| Name：XXX Phone：XXX  Email：XXX | Name：XXX Phone：XXX  Email：XXX |
| --- | --- |

**Volunteer statements:**

I make the following commitments and warranties:

1. I have read the detailed materials of the above project research carefully and understood the experimental procedure.
2. I answered all the questions about the experiment truthfully.
3. I know that the staff will evaluate me before the formal experiment to make sure that I am eligible to participate in the study.
4. I agree to comply with the requirements of the experiment, actively cooperate with the experiment, and insist on completing all experimental projects on time with both quality and quantity guaranteed. During the experiment, I will report any accident or abnormal situation immediately.
5. I understand that my name will not appear in the reports and papers related to the experiment, and I agree to use the research results in any way provided that confidentiality measures are taken.
6. I will keep confidential all the information I get during the experiment.

**Subject (volunteer)**

I declare that I have been informed of the purpose and process of this study and that all my questions have been satisfactorily answered, and that My signature below indicates that I wish to participate in this study.

Signature ________________

**Principal investigator**

I have explained the purpose of the study, the process, the potential hazards, and the subjects' rights and interests, and I have answered the questions related to the study to the maximum extent possible.

Signature ________________

Date________________
